# Phosphorylation and inactivation of PTEN at residues Ser380/Thr382/383 induced by *Helicobacter pylori* promotes gastric epithelial cell survival through PI3K/Akt pathway

**DOI:** 10.18632/oncotarget.5577

**Published:** 2015-09-10

**Authors:** Zhen Yang, Chuan Xie, Wenting Xu, Gongmeizi Liu, Ximei Cao, Wei Li, Jiang Chen, Yin Zhu, Shiwen Luo, Zhijun Luo, Nonghua Lu

**Affiliations:** ^1^ Department of Gastroenterology, The First Affiliated Hospital of Nanchang University, Nanchang, Jiangxi, China; ^2^ Center for Experimental Medicine, The First Affiliated Hospital of Nanchang University, Nanchang, Jiangxi, China; ^3^ The Medical College of Nanchang University, Nanchang, Jiangxi, China; ^4^ Department of Biochemistry, Boston University School of Medicine, Boston, MA, USA

**Keywords:** gastric carcinogenesis, Helicobacter pylori, phosphatase and tensin homolog, phosphorylation, PI3K/Akt pathway

## Abstract

Phosphorylation of PTEN at residues Ser380/Thr382/383 leads to loss of phosphatase activity and tumor suppressor function. Here, we found that phosphorylation of PTEN at residues Ser380/Thr382/383 was increased with gastric carcinogenesis, and more importantly, *Helicobacter pylori* was a trigger of this modification in chronic non-atrophic gastritis. *H. pylori* could phosphorylate and inactivate PTEN *in vivo* and *in vitro*, resulting in survival of gastric epithelial cells. Furthermore, stable expression of dominant-negative mutant PTEN or inhibition of Akt prevented the enhanced survival induced by *H. pylori*. These results indicate that PTEN phosphorylation at residues Ser380/Thr382/383 is a novel mechanism of PTEN inactivation in gastric carcinogenesis, and *H. pylori* triggers this modification, resulting in activation of the PI3K/Akt pathway and promotion of cell survival.

## INTRODUCTION

Gastric cancer is the fourth most common cancer worldwide, the third leading cause of cancer-related death in males and the fifth among females [1]. Although the incidence and mortality of gastric cancer have declined, it is still one of the most significant health burdens in the world [1, 2]. Treatment with chemotherapy has limited effects and usually serves as palliative care to relieve patient symptoms and increase survival time [1]. To date, surgical resection remains the only curative means to improve survival. As the underlying mechanisms responsible for the development and progression of gastric cancer are still poorly understood, further investigation is needed to develop novel preventative strategies. However, it is known that *Helicobacter pylori* infection induces chronic non-atrophic gastritis which can progress to intestinal metaplasia, dysplasia and finally gastric cancer [3]. Epidemiologic, animal and clinical studies have confirmed this critical role of *H. pylori*, which has been classified as a class I carcinogen for gastric cancer by the International Agency for Research of Cancer [4].

*H. pylori* infection is known to disturb the functions of some tumor suppressor genes and oncogenes in gastric tissue [5-10], which may be the initial carcinogenic trigger. Phosphatase and tensin homolog (*PTEN*), a tumor suppressor gene identified in 1997 [11-13], encodes a lipid and protein phosphatase that is involved in the regulation of a variety of signaling transduction pathways, including the PI3K/Akt pathway, which are critical in cell apoptosis, adhesion and mobility, as well as in the regulation of chromosomal stability [14-18]. PTEN is frequently inactivated in various human cancers [11, 12, 19-21], and several studies have indicated that PTEN is frequently inactivated in gastric cancers due to genetic or epigenetic changes, such as mutation, loss of heterozygosity, promoter hypermethylation and regulation of microRNA [22-26]. However, it remains to be defined whether other mechanisms account for inactivation of PTEN and how PTEN inactivation promotes carcinogenesis.

PTEN activity can be regulated by a variety of mechanisms, including acetylation, oxidation, ubiquitination, and most importantly, by phosphorylation [27-32]. Several phosphorylation sites have been identified in PTEN that lead to a loss of phosphatase activity or a gain of stability, such as Ser380, Thr382, and Thr383 [27, 28], which ultimately may result in loss of tumor suppressor function and increased cancer susceptibility. An increase in phosphorylation of this C-terminal serine-threonine cluster and PTEN inactivation is observed in T-cell acute lymphoblastic leukemia and adult T-cell leukemia-lymphoma [33, 34]. In a previous study, we demonstrated that aberrant phosphorylation of PTEN at residue Ser380 was an early event that could contribute to gastric carcinogenesis [21]. Therefore, the aim of the present study was to determine the expression and phosphorylation of PTEN at residues Ser380/Thr382/383 in gastric lesions of different stages with or without *H. pylori* infection. Furthermore, the effect of *H. pylori* infection on PTEN phosphorylation and activation of downstream PI3K/Akt pathway-related proteins was evaluated *in vitro* and *in vivo* in an effort to identify mechanisms of gastric carcinogenesis.

## RESULTS

### PTEN phosphorylation is increased in gastric cancer tissues

**Figure 1:**
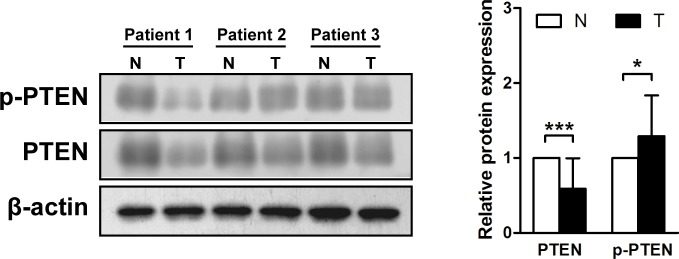
Phosphatase and tensin homolog is phosphorylated in gastric cancer tissues Immunoblots of phosphatase and tensin homolog (PTEN), phosphorylated (p)-PTEN and β-actin were used to quantify the relative protein expression levels in patient samples; densitometric units of gastric tumor samples (T) are expressed as fold of their non-cancerous counterparts (N). The samples derive from the same experiment and that blots were processed in parallel. *p < 0.05; ***p < 0.001.

Western blotting was performed on protein samples from gastric cancer tissues to determine if PTEN is phosphorylated at residues Ser380/Thr382/383. A reduction in PTEN expression was detected in 13 of the 15 (86.7%) primary gastric cancer samples (in a range of 0.07–1.46, mean = 0.59, p < 0.001), though ten cases (66.7%) showed an increase in the proportion of phosphorylated PTEN in gastric cancer tissues compared with the paired adjacent mucosa (in a range of 0.48–2.26, mean = 1.29, p < 0.05) (Figure 1).

### Phosphorylated PTEN in chronic non-atrophic gastritis is enhanced with *H. pylori* infection

**Figure 2:**
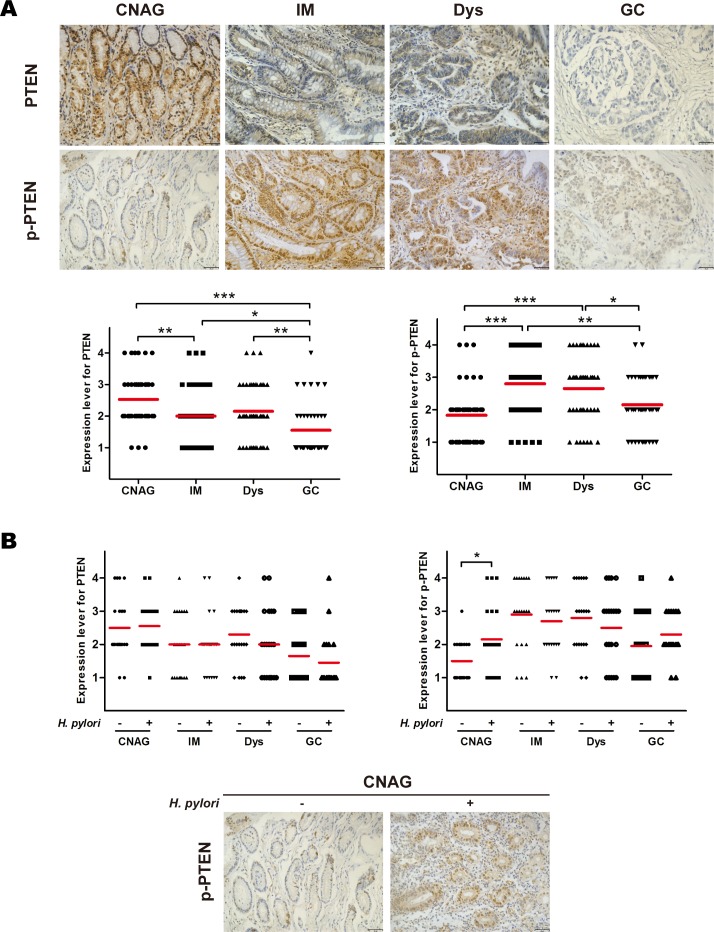
Phosphorylation of phosphatase and tensin homolog throughout the development of gastric cancer and in relation to *H. pylori* infection Gastric tissue samples were stained with antibodies against phosphatase and tensin homolog (PTEN) and phosphorylated (p)-PTEN; immunoreactive cells positive for PTEN and p-PTEN were semi-quantitatively assessed, and the protein expression levels are expressed as grade 1–4 in A: all patients in various stages of gastric lesions and B: patients in various stages of gastric lesions with or without *H. pylori* infection. Mean grades (−) for protein expression are shown. CNAG, chronic non-atrophic gastritis; IM, intestinal metaplasia; Dys, dysplasia; GC, gastric cancer. Scale bar = 40 μm. *p < 0.05; ***p < 0.001.

Immunohistochemistry was performed to evaluate the phosphorylation of PTEN throughout the various stages of gastric carcinogenesis. The results show that weak to strong expression of PTEN was observed in 92.5%, 65.0%, 70.0%, and 37.5% of chronic non-atrophic gastritis, intestinal metaplasia, dysplasia, and gastric cancer, respectively. PTEN expression level was significantly decreased in gastric cancer compared to chronic non-atrophic gastritis, intestinal metaplasia, or dysplasia (*ps* < 0.05) (Figure 2A). In addition, there was significant difference in PTEN expression between the tissue specimens of chronic non-atrophic gastritis and intestinal metaplasia (*p* < 0.01) (Figure 2A). Moreover, Weak to strong phosphorylated PTEN was observed in 57.5%, 87.5%, 82.5%, and 75.0% of chronic non-atrophic gastritis, intestinal metaplasia, dysplasia, and gastric cancer, respectively, indicating that phosphorylated PTEN was significantly increased in intestinal metaplasia, and dysplasia compared to chronic non-atrophic gastritis (*ps* < 0.001) (Figure 2A). Again, there was significant difference in phosphorylated PTEN between intestinal metaplasia or dysplasia and gastric cancer (*ps* < 0.05) (Figure 2A). Nevertheless, our data obtained from 25 cases of gastric cancer do not show an obvious association between PTEN or phosphorylated PTEN levels and clinicopathological grades (Table S1). These data suggest that phosphorylation of PTEN is an early event and may contribute to gastric carcinogenesis.

As it has been postulated that *H. pylori* infection may trigger gastric carcinogenesis, the same cohort of patients were divided to *H. pylori*-positive and -negative two groups, and the phosphorylation of PTEN was examined in patients with or without *H. pylori* infection. Immunohistochemical analysis revealed that infection with *H. pylori* did not affect the loss of PTEN expression, however, the phosphorylated PTEN was significantly increased by infection in chronic non-atrophic gastritis (*p* < 0.05) (Figure 2B). These results suggest that *H. pylori* infection enhances or accelerates the phosphorylation of PTEN.

### *H. pylori* infection induces phosphorylation of PTEN and activation of the PI3K/Akt pathway in gastric tissue from Mongolian gerbils

The Mongolian gerbils were successfully infected with *H. pylori* which was confirmed by Giemsa staining, no animals challenged with Brucella broth alone had detectable evidence of *H. pylori*.

**Figure 3:**
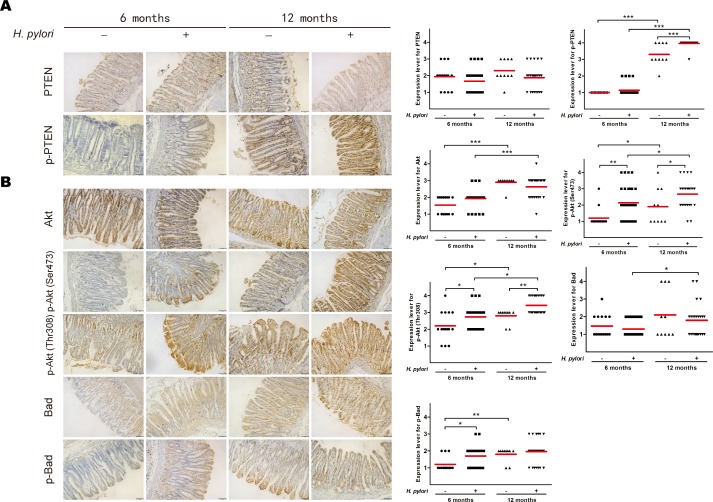
*H. pylori* infection induces phosphorylation of phosphatase and tensin homolog and PI3K/Akt pathway-related proteins in gastric tissue of Mongolian gerbils Gastric tissue sections from *H. pylori-*infected gerbils were taken after 6 or 12 months and stained for antibodies against A: phosphatase and tensin homolog (PTEN) and phosphorylated (p)-PTEN and B: Akt, p-Akt, Bad and p-Bad. Immunoreactive cells were semi-quantitatively assessed, and the protein expression levels are expressed as grade 1–4. Mean grades (−) for protein expression are shown. Scale bar = 40 μm. *p < 0.05; **p < 0.01; ***p < 0.001.

To confirm that *H. pylori* infection induces phosphorylation of PTEN and assess downstream activation *in vivo*, immunohistochemical analysis of PTEN and PI3K/Akt pathway-related proteins (*i.e.*, Akt, phosphorylated Akt [active phosphorylation of Akt], Bad, and phosphorylated Bad [inhibitory phosphorylation of Bad]) was performed from gastric tissue samples of Mongolian gerbils with or without *H. pylori* infection after 6 and 12 months. Although the expression levels of PTEN, Akt, and Bad did not differ, the levels of phosphorylated PTEN, phosphorylated Akt, and phosphorylated Bad were higher 6 and/or 12 months after *H. pylori* infection (*ps* < 0.05) (Figure 3). Interestingly, the levels of phosphorylated PTEN and all downstream PI3K/Akt pathway-related proteins was higher in Mongolian gerbils sacrificed at 12 months than those sacrificed at 6 months regardless of *H. pylori* infection, suggesting pathway activation with age.

### *H. pylori* induces phosphorylation of PTEN and activation of the PI3K/Akt pathway *in vitro*

**Figure 4:**
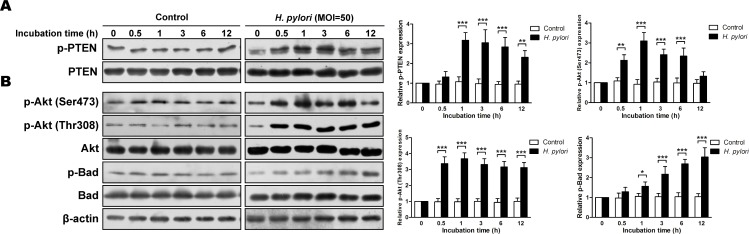
*H. pylori* induces phosphorylation of phosphatase and tensin homolog and PI3K/Akt pathway-related proteins *in vitro* Cultures of gastric epithelial cells (GES-1) were incubated for various times with *H. pylori* (multiplicity of infection [MOI] = 50) in *H. pylori* group or with medium in control group. Immunoblots of A: phosphatase and tensin homolog (PTEN) and phosphorylated (p)-PTEN and B: Akt, p-Akt, Bad, p-Bad and β-actin were used to quantify the relative protein expression levels (expressed as fold of control). The data are representative of three independent experiments. The samples derive from the same experiment and that blots were processed in parallel. *p < 0.05; **p < 0.01; ***p < 0.001.

Incubation of non-malignant GES-1 cells with *H. pylori* resulted in a significant increase in the proportion of phosphorylated PTEN as compared with a control (*ps* < 0.01) (Figure 4A). In addition, the proportions of phosphorylated Akt (Ser473 and Thr308) and Bad were increased with *H. pylori* incubation (*ps* < 0.05) (Figure 4B). The increased phosphorylation of these proteins demonstrated a dose dependency (at 1h) (Figure S1), suggesting that *H. pylori* also induces phosphorylation of PTEN and activation the PI3K/Akt pathway *in vitro*.

### Activation of the PI3K/Akt pathway by PTEN regulation

**Figure 5:**
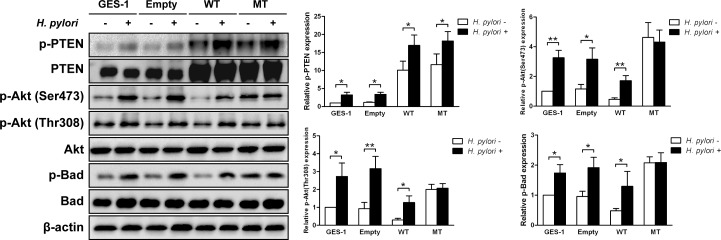
*H. pylori*-induced phosphorylation P13K/Akt pathway-related proteins is blocked in cells expressing mutant phosphatase and tensin homolog Immunoblots of phosphatase and tensin homolog (PTEN), phosphorylated (p)-PTEN, Akt, p-Akt, Bad, p-Bad and β-actin from gastric epithelial cells (GES-1) stably expressing an empty vector (Empty), wild-type (WT) or mutant dominant-negative phosphatase and tensin homolog (C124S) (MT) were used to quantify the relative protein expression levels after a 1 h incubation with *H. pylori* (multiplicity of infection = 50) (expressed as fold of control). The data are representative of three independent experiments. The samples derive from the same experiment and that blots were processed in parallel. *p < 0.05; **p < 0.01.

To determine the role of PTEN regulation on *H. pylori*–induced activation of the PI3K/Akt pathway, stable cell lines expressing a wild-type or dominant-negative mutant PTEN (C124S) were tested. PTEN was overexpressed in stable cell line carried with the wild-type PTEN which display its phosphatase activity soundly. However, a dominant-negative mutant PTEN of C124S was the catalytically dead mutant form of the phosphatase. Phosphorylation and inactivation of PTEN at residues Ser380/Thr382/383 were mimiced in stable cell line carried with a dominant-negative mutant PTEN. Further, the empty vector was not carried with any exogenous gene. As expected, *H. pylori* (multiplicity of infection [MOI] = 50 for 1h) resulted in significant increases in the phosphorylation of PTEN, Akt, and Bad in the stable cell line expressing the wild-type PTEN, similar to that observed in GES-1 cells and a stable cell line expressing an empty vector (all *ps* < 0.05) (Figure 5). However, there was an increase in the proportion of phosphorylated PTEN with *H. pylori*, but no difference in the phosphorylation of Akt or Bad in the stable cell line expressing the mutant PTEN. These data suggest that a mutant inactive PTEN mimics *H. pylori*-induced activation of the PI3K/Akt pathway.

However, *H. pylori* has been reported to activate PI3K directly [35-40]. In present study, inhibition of PI3K with LY294002 did not affect phosphorylation of PTEN, but diminish phosphorylation of Akt or Bad upon incubation with *H. pylori* (MOI = 50 for 1h) or not (Figure S2). In addition, Akt Inhibitor VIII blocked the *H. pylori*-induced phosphorylation of Akt and Bad, but not PTEN (MOI = 50 for 1h) (Figure S3). Therefore, these results suggested that the mechanisms of activation of Akt by *H. pylori* involve direct activation of PI3K and phosphorylation and inactivation of PTEN.

### *H. pylori* promotes gastric epithelial cell viability through the PTEN/Akt pathway

**Figure 6:**
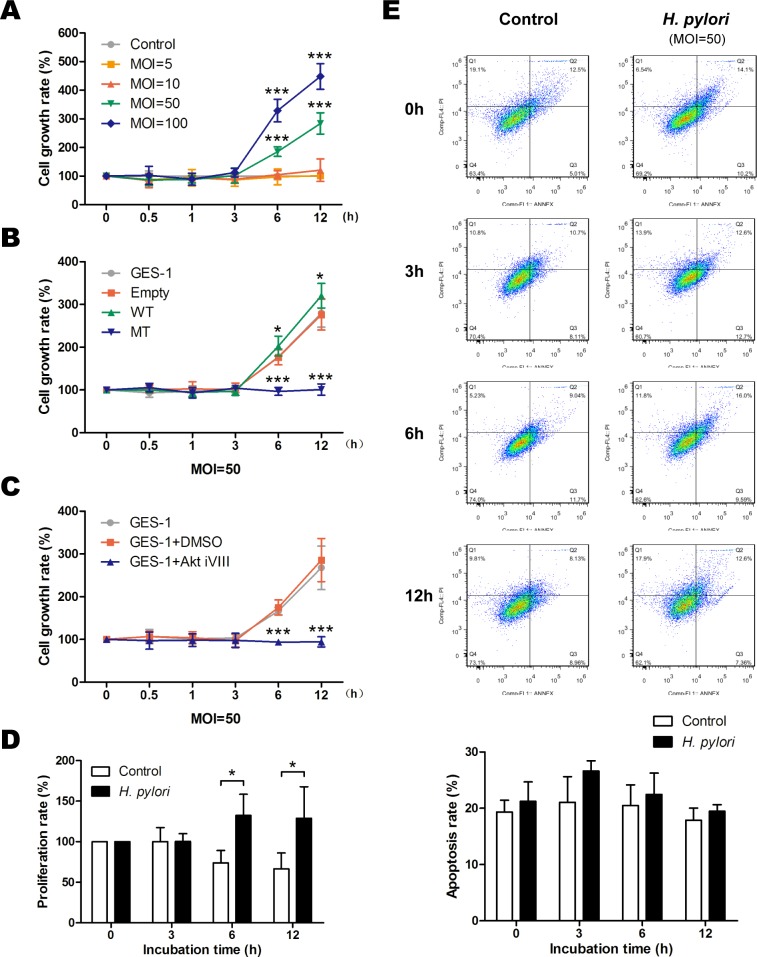
*H. pylori* promotes gastric epithelial cell survival through phosphorylation of phosphatase and tensin homolog and activation of Akt pathway Cell survival was assessed using a modified MTT assay, and the cell survival rate was expressed as percentage of control or gastric epithelial cells (GES-1) at different time points after A: incubation with various doses (multiplicity of infection [MOI]) of *H. pylori*, B: in cells stably expressing an empty vector (Empty, wild-type (WT) or mutant dominant-negative phosphatase and tensin homolog (C124S) (MT), or C: after incubation with an inhibitor of Akt. D: Proliferation in control or *H. pylori-*infected cells was assessed using a BrdU assay, and the proliferation rate was calculated as the percentage of control or *H. pylori-*infected cells at 0 h. E: Apoptosis in control or *H. pylori-*infected cells was assessed using a flow cytometry analysis, and the apoptosis rate was calculated as the ratio of “Q2 + Q3” to the total cell number. The data are representative of three independent experiments. *p < 0.05; ***p < 0.001.

Aberrant activation of the Akt pathway induced by *H. pylori* infection may affect cell viability. Evaluation of cell viability using an MTT assay showed that *H. pylori* increased the growth rate of GES-1 cells in both a time- and dose-dependent manner (Figure 6A), and the proliferation rates were also increased as determined by a BrdU assay (Figure 6D); However, apoptosis rates were not affected as determined by flow cytometry analysis (MOI = 50) (Figure 6E). The *H. pylori*-induced increased growth was blocked by mutant PTEN C124S expression (Figure 6B) and Akt inhibition (Figure 6C). A similar effect was observed *in vivo*, as *H. pylori* infection increased cell proliferation in gastric tissue after 6 months as determined by proliferation cell nuclear antigen (PCNA) immunostaining (Figure S4). This difference was not apparent after 12 months of infection, likely masked by the increased proliferation observed in both groups at 12 months compared to 6 months. The increased proliferation was not a result of decreased apoptosis, as there was no difference among the groups as determined by a TUNEL assay. Taken together, these results strongly suggest that phosphorylation and inactivation of PTEN induced by *H. pylori* promotes gastric epithelial cell survival through the Akt pathway.

## DISCUSSION

Although it has been suggested that PTEN is vital to gastric carcinogenesis, data supporting this conjecture remain mostly limited to associations with genetic or epigenetic changes [22-26]. However, PTEN inactivation through increased phosphorylation of its C-terminal serine-threonine cluster is observed in some hematopoietic tumors [33, 34]. In a previous study, we demonstrated that aberrant phosphorylation of PTEN at residue Ser380 is an early event that could contribute to gastric carcinogenesis [21]. The results of the present study show that PTEN phosphorylation at Ser380/Thr382/383 is abnormally increased in gastric cancer tissues.

The development of gastric cancer is considered a multistep process beginning with chronic non-atrophic gastritis, and progressing through intestinal metaplasia and dysplasia to gastric cancer [3]. The data show that this progression is paralleled by a loss of PTEN, and taking into account total PTEN reduction, an increase in phosphorylated PTEN, yielding a sustained high expression in intestinal metaplasia, dysplasia and gastric cancer. Additionally, the results revealed that the phosphorylated PTEN was significantly higher in patients with chronic non-atrophic gastritis that were infected with *H. pylori*, but not at later stages of gastric carcinogenesis. Although *H. pylori* is a class I carcinogen for gastric cancer [4], it is one of risk factors for gastric cancer and is often combined with other factors, such as genetics, the environment, diet, and socio-economic status to jointly lead to gastric cancer. Taking into account the result that phosphorylated PTEN is progressively increased in gastric carcinogenesis regardless of *H. pylori* infection, we speculate that not only *H. pylori* infection, but also other risk factors may induce phosphorylation of PTEN in gastric carcinogenesis, however, *H. pylori* infection could accelerate the phosphorylation of PTEN in the early stages of gastric carcinogenesis. And further studies incorporating different risk factors are needed. In addition, the effect of *H. pylori* on PTEN phosphorylation was further demonstrated both *in vivo* and *in vitro*. These results further support the role of PTEN phosphorylation in gastric carcinogenesis, and suggest that *H. pylori* infection accelerates this process.

The PI3K/Akt pathway is downstream of PTEN [14] and is frequently activated in gastric carcinogenesis and vital to gastric cancer development [41]. This pathway was found to be activated by *H. pylori* infection both *in vivo* and *in vitro*, which is consistent with previous studies [9, 10, 35-40, 42]. Interference with PTEN activity with a dominant-negative mutant inhibits the effect of *H. pylori* on the Akt pathway, and suppressing Akt blocked the increase in cell survival, which suggest that the mechanisms of activation of Akt by *H. pylori* involve direct activation of PI3K and phosphorylation and inactivation of PTEN. Aberrant PI3K/Akt pathway signaling is the underlying defect found in several pathologies. Akt is one of the most frequently activated kinases in human cancer and can promote unregulated cell proliferation. Based on the data observed in the present study, we conclude that *H. pylori* infection leads to phosphorylation of PTEN at residues Ser380/Thr382/383 and aberrant activation of the PI3K/Akt pathway, resulting in increased cell survival both *in vivo* and *in vitro*, which is consistent with previous work [5, 36].

Although these results enhance our understanding of gastric carcinogenesis with relation to *H. pylori* infection, there are a few limitations in the present study. For example, the study was based only on a CagA^+^ and VagA^+^ strain of *H. pylori*, and the virulence factors responsible for phosphorylation of PTEN at residues Ser380/Thr382/383 are still unknown. Further, the study only focused on inactivation of lipid phosphatase activity of PTEN and not regulation of other downstream factors, such as focal adhesion kinase [15, 17].

In addition, there is a discrepancy in this study. In present study, we found that *H. pylori* promotes gastric epithelial cell proliferation *in vitro*, which was different from our previous study that *H. pylori* filtrates have cytostatic and cytotoxic effects on gastric cells by inducing DNA damage, G1/S cell cycle arrest, and apoptosis [9]. Indeed, increased proliferation and reduced survival of *H. pylori*-infected cells have been reported previously [43]. These seemingly opposing results reveal the diverse effects of *H. pylori* infection that could often be reconciled by differences in experimental models [43]. An interesting example is a recent study by Wroblewski *et al*., who have provided evidence that the *H. pylori* strains which inhibit growth of gastric cells in a co-culture model induce cellular proliferation in gastric organoids [44]. Therefore, further studies incorporating different models are needed.

In conclusion, this study indicates that reduced expression of PTEN and increased PTEN phosphorylation at residues Ser380/Thr382/383 could contribute to gastric carcinogenesis, and targeting phosphorylation at these sites could be a novel mechanism for treating gastric carcinogenesis. Moreover, *H. pylori* accelerates this phosphorylation, which activates the PI3K/Akt pathway and promotes cell survival. Future clinical and experimental studies will help to elucidate the mechanism by which *H. pylori* induces PTEN phosphorylation, and how this advances gastric carcinogenesis.

## MATERIALS AND METHODS

### Patients and tissue specimens

Gastric adenocarcinoma and corresponding non-cancerous tissue samples were collected during surgery from 15 patients who were not receiving any adjuvant therapy between January 2012 and June 2012 at The First Affiliated Hospital of Nanchang University. An additional 160 gastric tissue samples were collected from patients who underwent a gastroduodenoscopy at the same hospital between January 2007 and September 2009, including 40 cases of chronic non-atrophic gastritis (20 *H. pylori*-positive, 20 negative), 40 cases of intestinal metaplasia (20 *H. pylori*-positive, 20 negative), 40 cases of dysplasia (20 *H. pylori*-positive, 20 negative), and 40 gastric cancers (20 *H. pylori*-positive, 20 negative). There were no significant differences in the age or gender distribution among these groups, which are summarized in Table S2. Pathologic diagnosis and classification were made according to the criteria of the World Health Organization [45] and the updated Sydney system [46]. Detection of *H. pylori* infection was carried out using a rapid urease test and Giemsa staining as previously described [5-9]. Informed consent was obtained from all patients, all procedures were approved and carried out in accordance with the guidelines by the Ethics Committee of The First Affiliated Hospital of Nanchang University.

### Cell line and *H. pylori* strain

The immortalized human gastric epithelial mucosa cell line GES-1 was cultured in Dulbecco's Modified Eagle Medium (DMEM) supplemented with 10% fetal bovine serum (FBS), 100 U penicillin, and 100 μg/ml streptomycin (Gibco of Thermo Fisher Scientific Inc., Waltham, MA, USA) at 37°C in an atmosphere of 5% CO_2_. The CagA^+^ and VagA^+^ *H. pylori* type strain ATCC43504 was cultured on Campylobacter agar plates containing 10% sheep serum and incubated at 37°C under microaerophilic conditions (5% O_2_, 10% CO_2_, and 85% N_2_) for 24 h, then subcultured in Brucella broth supplemented with 10% FBS at 37°C under microaerophilic atmosphere for 16-18 h. Bacterial density was estimated spectrophotometrically as absorbance at 600 nm (OD_600_), and viable counts were determined as colony-forming units (CFU)⁄ml (1 OD_600_ = 10^9^ CFU/ml).

### Mongolian gerbils

Five to eight weeks old specific-pathogen-free male Mongolian gerbils (30-50 g) provided by Zhejiang Academy of Medical Sciences (Hangzhou, Zhejiang, China) were maintained in an isolated clean room with regulated temperature (20–22°C), humidity (approximately 55%), and a 12/12-h light/dark cycle, with ad libitum rodent diet and water. After one week's observation, gerbils were given orogastric infusions of 1ml either sterile Brucella broth (*n* = 25) or 1 × 10^8^ CFU of *H. pylori* type strain ATCC43504 (*n* = 54) once every 3 days for a total of ten infusions. Gerbils were fasted for 12 h before, and drinking water was withheld after *H. pylori* inoculation. Both food and water were then freely available to animals 4 h after inoculation. Animals were euthanized at either 6 months (*n* = 30 *H. pylori*-infected; 15 controls) or 12 months (*n* = 24 *H. pylori*-infected; 10 controls), and linear strips of gastric tissue extending from the squamocolumnar junction through the proximal duodenum were collected. All protocols were approved and carried out in accordance with the guidelines by the Ethics Committee of The First Affiliated Hospital of Nanchang University.

### Reagents and lentivirus

Pharmacological inhibition of PI3K was achieved with LY294002 (40 μM; Sigma-Aldrich, St. Louis, MO, USA), and Akt was inhibited with Akt Inhibitor VIII (10 μM; Merck KGaA, Darmstadt, Germany). Wild-type PTEN, dominant-negative mutant type PTEN (C124S), and empty lentiviral supernatants were purchased from Invitrogen (of Thermo Fisher Scientific Inc.). For lentivirus infection, GES-1 cells were grown to approximately 60% confluence and incubated with viral supernatants and hexadimethrine bromide (Sigma-Aldrich, St. Louis, MO, USA) for 6 h. Forty-eight hours later, the cells were split and cultured in selection media containing blasticidin (Sigma-Aldrich) for an additional 2 weeks. Then the stable cell lines expressing a wild-type or dominant-negative mutant PTEN were established.

### Immunoblotting

Western blotting was performed according to standard methods as described previously [21] using anti-PTEN (#9559; 1:10000), anti-phosphorylated (p)-PTEN (Ser380/Thr382/383) (#9554; 1:10000), anti-Akt (#4691; 1:10000), anti-phosphorylated (p)-Akt (Ser473) (#9271; 1:1000), anti-phosphorylated (p)-Akt (Thr308) (#9275; 1:1000), anti-Bad (#4366; 1:1000), anti-phosphorylated (p)-Bad (Ser136) (#4366; 1:1000) antibodies (Cell Signaling Technology, Danvers, MA, USA), and an anti-β-actin antibody (sc-1615-R; 1:1000; Santa Cruz Biotechnology, Dallas, TX, USA).

### Immunohistochemistry

Immunohistochemistry was performed on paraffin sections of human biopsy specimens or Mongolian gerbil gastric tissues using anti-PTEN (ab31392; 1:150 [biopsy], 1:800 [gastric tissues]), anti-p-PTEN (Ser380/Thr382/383) (ab47332; 1:800 [biopsy], 1:1000 [gastric tissues]), anti-Akt (ab8805; 1:400), anti-p-Akt (Ser473) (ab66138; 1:400), anti-p-Akt (Thr308) (ab38449; 1:200), anti-Bad (ab32445; 1:1000), and anti-p-Bad (Ser136) (ab28824; 1:1000) antibodies (Abcam, Cambridge, UK) following previously described methods [6-9, 21], the specificity of the staining of different antibodies was evaluated by corresponding blocking peptides, and the negative control sections were just incubated with PBS without the primary antibodies. The stained sections were chosen, reviewed, and scored from five randomly selected high power fields (40× objective lens) by two pathologists blinded to the histopathologic data. Grading discrepancies were re-reviewed and discussed to obtain a final score. Epithelial cells with yellow or brown staining in the nucleus and/or cytoplasm was defined as positive for immunoreactivity. The percentage of immunoreactive cells from 100 cells in each field were averaged from the five fields and scored as follows: 0 < 5.0% immunoreactive; 1 = 5.1–25.0%; 2 = 25.1–50.0%; 3 = 50.1–75.0%; and 4 > 75.0%. Moreover, the staining intensity was also semi-quantitatively assessed as 0 = no staining; 1 = weak staining; 2 = moderate staining; and 3 = strong staining. The overall protein expression level was then reported as a grade as calculated from an integral score of the “area × intensity” as follows: grade 1 = score 0–2 (negative); grade 2 = score 3–5 (weakly positive); grade 3 = score 6–8 (moderately positive); and grade 4 = score 9–12 (strongly positive).

### MTT assay, BrdU and flow cytometry analysis

Cell survival or proliferation was assessed using a modified MTT assay or a BrdU cell proliferation assay kit (EMD Millipore, Billerica, MA, USA) as described previously [47]. Apoptosis in cell lines was determined by using a flow cytometry analysis (Annexin V: FITC Apoptosis Detection Kit I; Becton, Dickenson and Company, Franklin Lakes, NJ, USA) according to the manufacturer's instructions.

### Statistical analysis

Data are summarized as mean ± standard deviation (SD) or percentage of control. The chi-square test was performed to evaluate differences in categorical variables, such as gender, among different defined groups. The one-way analysis of variance (ANOVA) was used to determine the differences in numerical variables, such as patients' ages, among the groups. Kruskal-Wallis or Mann-Whitney tests were used to determine the differences in numerical variables between differently defined groups. A p value of < 0.05 was considered as statistically significant.

## SUPPLEMENTARY MATERIAL FIGURES AND TABLES



## References

[1] American Cancer Society (2011). Global Cancer Facts & Figures.

[2] Bertuccio P, Chatenoud L, Levi F, Praud D, Ferlay J, Negri E, Malvezzi M, La Vecchia C (2009). Recent patterns in gastric cancer: a global overview. Int J Cancer.

[3] Correa P (1992). Human gastric carcinogenesis: a multistep and multifactorial process--First American Cancer Society Award Lecture on Cancer Epidemiology and Prevention. Cancer Res.

[4] Schistosomes, liver flukes and Helicobacter pylori (1994). IARC Working Group on the Evaluation of Carcinogenic Risks to Humans. Lyon, 7-14 June 1994. IARC Monogr Eval Carcinog Risks Hum.

[5] Zhu Y, Shu X, Chen J, Xie Y, Xu P, Huang DQ, Lu NH (2008). Effect of Helicobacter pylori eradication on oncogenes and cell proliferation. Eur J Clin Invest.

[6] Yang Z, Shu X, Chen L, Chen J, Xie Y, Lu NH (2012). Expression of p53-MDM2 feedback loop related proteins in different gastric pathologies in relation to Helicobacter pylori infection: implications in gastric carcinogenesis. Clin Res Hepatol Gastroenterol.

[7] Li W, Xie C, Yang Z, Chen J, Lu NH (2013). Abnormal DNA-PKcs and Ku 70/80 expression may promote malignant pathological processes in gastric carcinoma. World J Gastroenterol.

[8] Xie C, Xu LY, Yang Z, Cao XM, Li W, Lu NH (2014). Expression of gammaH2AX in various gastric pathologies and its association with infection. Oncol Lett.

[9] Shu X, Yang Z, Li ZH, Chen L, Zhou XD, Xie Y, Lu NH (2015). Helicobacter pylori Infection Activates the Akt-Mdm2-p53 Signaling Pathway in Gastric Epithelial Cells. Dig Dis Sci.

[10] Wei J, Nagy TA, Vilgelm A, Zaika E, Ogden SR, Romero-Gallo J, Piazuelo MB, Correa P, Washington MK, El-Rifai W, Peek RM, Zaika A (2010). Regulation of p53 tumor suppressor by Helicobacter pylori in gastric epithelial cells. Gastroenterology.

[11] Li J, Yen C, Liaw D, Podsypanina K, Bose S, Wang SI, Puc J, Miliaresis C, Rodgers L, McCombie R, Bigner SH, Giovanella BC, Ittmann M (1997). PTEN, a putative protein tyrosine phosphatase gene mutated in human brain, breast, and prostate cancer. Science.

[12] Steck PA, Pershouse MA, Jasser SA, Yung WK, Lin H, Ligon AH, Langford LA, Baumgard ML, Hattier T, Davis T, Frye C, Hu R, Swedlund B (1997). Identification of a candidate tumour suppressor gene, MMAC1, at chromosome 10q23. 3 that is mutated in multiple advanced cancers. Nat Genet.

[13] Li DM, Sun H (1997). TEP1, encoded by a candidate tumor suppressor locus, is a novel protein tyrosine phosphatase regulated by transforming growth factor beta. Cancer Res.

[14] Stambolic V, Suzuki A, de la Pompa JL, Brothers GM, Mirtsos C, Sasaki T, Ruland J, Penninger JM, Siderovski DP, Mak TW (1998). Negative regulation of PKB/Akt-dependent cell survival by the tumor suppressor PTEN. Cell.

[15] Tamura M, Gu J, Matsumoto K, Aota S, Parsons R, Yamada KM (1998). Inhibition of cell migration, spreading, and focal adhesions by tumor suppressor PTEN. Science.

[16] Gu J, Tamura M, Yamada KM (1998). Tumor suppressor PTEN inhibits integrin- and growth factor-mediated mitogen-activated protein (MAP) kinase signaling pathways. J Cell Biol.

[17] Tamura M, Gu J, Takino T, Yamada KM (1999). Tumor suppressor PTEN inhibition of cell invasion, migration, and growth: differential involvement of focal adhesion kinase and p130Cas. Cancer Res.

[18] Shen WH, Balajee AS, Wang J, Wu H, Eng C, Pandolfi PP, Yin Y (2007). Essential role for nuclear PTEN in maintaining chromosomal integrity. Cell.

[19] Sansal I, Sellers WR (2004). The biology and clinical relevance of the PTEN tumor suppressor pathway. J Clin Oncol.

[20] Yang Z, Yuan XG, Chen J, Lu NH (2012). Is NEDD4-1 a negative regulator of phosphatase and tensin homolog in gastric carcinogenesis?. World J Gastroenterol.

[21] Yang Z, Yuan XG, Chen J, Luo SW, Luo ZJ, Lu NH (2013). Reduced expression of PTEN and increased PTEN phosphorylation at residue Ser380 in gastric cancer tissues: a novel mechanism of PTEN inactivation. Clin Res Hepatol Gastroenterol.

[22] Wang JY, Huang TJ, Chen FM, Hsieh MC, Lin SR, Hou MF, Hsieh JS (2003). Mutation analysis of the putative tumor suppressor gene PTEN/MMAC1 in advanced gastric carcinomas. Virchows Arch.

[23] Byun DS, Cho K, Ryu BK, Lee MG, Park JI, Chae KS, Kim HJ, Chi SG (2003). Frequent monoallelic deletion of PTEN and its reciprocal associatioin with PIK3CA amplification in gastric carcinoma. Int J Cancer.

[24] Kang YH, Lee HS, Kim WH (2002). Promoter methylation and silencing of PTEN in gastric carcinoma. Lab Invest.

[25] Guo J, Miao Y, Xiao B, Huan R, Jiang Z, Meng D, Wang Y (2009). Differential expression of microRNA species in human gastric cancer versus non-tumorous tissues. J Gastroenterol Hepatol.

[26] Xu WT, Yang Z, Lu NH (2014). Roles of PTEN (Phosphatase and Tensin Homolog) in Gastric Cancer Development and Progression. Asian Pac J Cancer Prev.

[27] Vazquez F, Ramaswamy S, Nakamura N, Sellers WR (2000). Phosphorylation of the PTEN tail regulates protein stability and function. Mol Cell Biol.

[28] Torres J, Pulido R (2001). The tumor suppressor PTEN is phosphorylated by the protein kinase CK2 at its C terminus. Implications for PTEN stability to proteasome-mediated degradation. J Biol Chem.

[29] Okumura K, Mendoza M, Bachoo RM, DePinho RA, Cavenee WK, Furnari FB (2006). PCAF modulates PTEN activity. J Biol Chem.

[30] Chae HD, Broxmeyer HE (2011). SIRT1 deficiency downregulates PTEN/JNK/FOXO1 pathway to block reactive oxygen species-induced apoptosis in mouse embryonic stem cells. Stem Cells Dev.

[31] Wang X, Trotman LC, Koppie T, Alimonti A, Chen Z, Gao Z, Wang J, Erdjument-Bromage H, Tempst P, Cordon-Cardo C, Pandolfi PP, Jiang X (2007). NEDD4-1 is a proto-oncogenic ubiquitin ligase for PTEN. Cell.

[32] Xu W, Yang Z, Zhou SF, Lu N (2014). Posttranslational regulation of phosphatase and tensin homolog (PTEN) and its functional impact on cancer behaviors. Drug Des Devel Ther.

[33] Silva A, Yunes JA, Cardoso BA, Martins LR, Jotta PY, Abecasis M, Nowill AE, Leslie NR, Cardoso AA, Barata JT (2008). PTEN posttranslational inactivation and hyperactivation of the PI3K/Akt pathway sustain primary T cell leukemia viability. J Clin Invest.

[34] Nakahata S, Ichikawa T, Maneesaay P, Saito Y, Nagai K, Tamura T, Manachai N, Yamakawa N, Hamasaki M, Kitabayashi I, Arai Y, Kanai Y, Taki T (2014). Loss of NDRG2 expression activates PI3K-AKT signalling via PTEN phosphorylation in ATLL and other cancers. Nat Commun.

[35] Sokolova O, Bozko PM, Naumann M (2008). Helicobacter pylori suppresses glycogen synthase kinase 3beta to promote beta-catenin activity. J Biol Chem.

[36] Suzuki M, Mimuro H, Kiga K, Fukumatsu M, Ishijima N, Morikawa H, Nagai S, Koyasu S, Gilman RH, Kersulyte D, Berg DE, Sasakawa C (2009). Helicobacter pylori CagA phosphorylation-independent function in epithelial proliferation and inflammation. Cell Host Microbe.

[37] Nagy TA, Frey MR, Yan F, Israel DA, Polk DB, Peek RM (2009). Helicobacter pylori regulates cellular migration and apoptosis by activation of phosphatidylinositol 3-kinase signaling. J Infect Dis.

[38] Nakayama M, Hisatsune J, Yamasaki E, Isomoto H, Kurazono H, Hatakeyama M, Azuma T, Yamaoka Y, Yahiro K, Moss J, Hirayama T (2009). Helicobacter pylori VacA-induced inhibition of GSK3 through the PI3K/Akt signaling pathway. J Biol Chem.

[39] Wen S, So Y, Singh K, Slingerland JM, Resnick MB, Zhang S, Ruiz V, Moss SF (2012). Promotion of cytoplasmic mislocalization of p27 by Helicobacter pylori in gastric cancer. Oncogene.

[40] Tabassam FH, Graham DY, Yamaoka Y (2012). Helicobacter pylori-associated regulation of forkhead transcription factors FoxO1/3a in human gastric cells. Helicobacter.

[41] Zhou XD, Chen HX, Guan RN, Lei YP, Shu X, Zhu Y, Lv NH (2012). Protein kinase B phosphorylation correlates with vascular endothelial growth factor A and microvessel density in gastric adenocarcinoma. J Int Med Res.

[42] Slomiany BL, Slomiany A (2011). Helicobacter pylori Induces Disturbances in Gastric Mucosal Akt Activation through Inducible Nitric Oxide Synthase-Dependent S-Nitrosylation: Effect of Ghrelin. ISRN Gastroenterol.

[43] Shirin H, Moss SF (1998). Helicobacter pylori induced apoptosis. Gut.

[44] Wroblewski LE, Piazuelo MB, Chaturvedi R, Schumacher M, Aihara E, Feng R, Noto JM, Delgado A, Israel DA, Zavros Y, Montrose MH, Shroyer N, Correa P, Wilson KT, Peek RM (2015). Helicobacter pylori targets cancer-associated apical-junctional constituents in gastroids and gastric epithelial cells. Gut.

[45] Hamilton SR, Aaltonen LA (2000). Pathology and genetics of tumors of the digestive system. World Health Organization classification of tumors.

[46] Dixon MF, Genta RM, Yardley JH, Correa P (1996). Classification and grading of gastritis. The updated Sydney System. International Workshop on the Histopathology of Gastritis, Houston 1994. Am J Surg Pathol.

[47] Yan R, Peng X, Yuan X, Huang D, Chen J, Lu Q, Lv N, Luo S (2013). Suppression of growth and migration by blocking the Hedgehog signaling pathway in gastric cancer cells. Cell Oncol (Dordr).

