# Supplementary material for: Phosphorylation and inactivation of PTEN at residues Ser380/Thr382/383 induced by Helicobacter pylori promotes gastric epithelial cell survival through PI3K/Akt pathway
Source: Oncotarget. 2015 Sep 10;6(31):31916–26. doi: 10.18632/oncotarget.5577 (PMC4741650; doi:10.18632/oncotarget.5577)
Supplement: Supplementary file 1 [file oncotarget-06-31916-s001.pdf]

Phosphorylation and inactivation of PTEN at residues Ser380/Thr382/383 induced by *Helicobacter pylori* promotes gastric epithelial cell survival through PI3K/Akt pathway

Supplementary Material

Epithelial cell proliferation and apoptosis

The proliferation of gastric epithelial cells was determined by proliferation cell nuclear antigen (PCNA) (ab29; 1:1800; Abcam, Cambridge, UK) immunohistochemistry in gastric tissue sections as previously described [5, 47]. Cells immunoreactive for PCNA in both superficial and glandular epithelium of corpus and antrum were semi-quantitatively assessed, and the protein expression levels were expressed as grade 1–4 as described in Materials and Methods. Apoptotic cells in the gastric epithelium was determined by using a TUNEL assay (DeadEnd Colorimetric TUNEL System; Promega Corp., Madison, WI, USA) according to the manufacturer’s instructions. The number of TUNEL positive epithelial cells was counted in the corpus and antrum. In both areas, approximately 1000 cells were counted. The epithelial apoptosis rate was calculated as the ratio of TUNEL positive cells to the total cell number as described previously [47]. The number of apoptotic epithelial cells in the gastric mucosa was determined for both the superficial epithelium and the glandular epithelium.

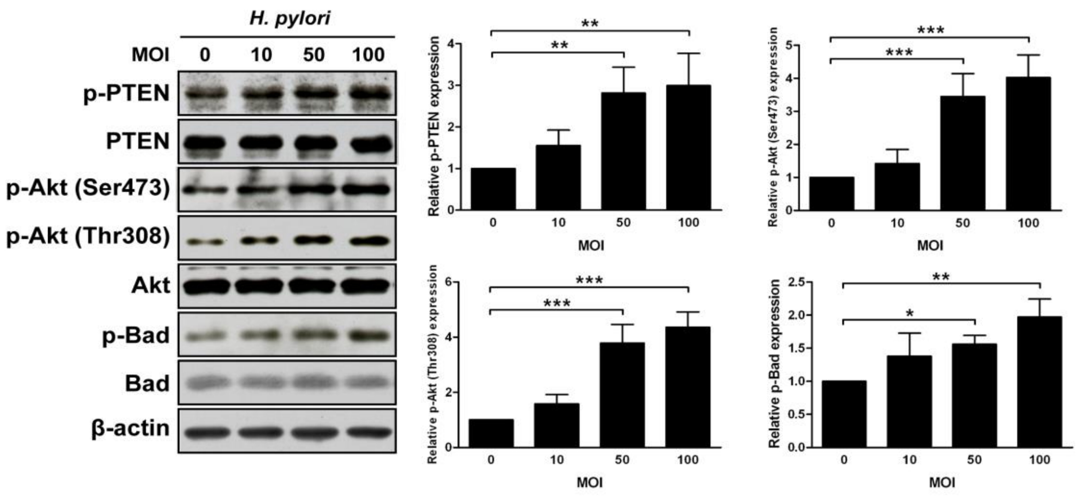

Figure S1. Phosphatase and tensin homolog is phosphorylated and PI3K/Akt pathway is activated by *H. pylori in vitro*. Immunoblots of phosphatase and tensin homolog/PI3K/Akt pathway related proteins were used to quantify the relative protein expression levels (expressed as fold of control) in gastric epithelial cells infected with *H. pylori* for 1 h. The data are representative of three independent experiments. The samples derive from the same experiment and that blots were processed in parallel. *$p < 0.05$; **$p < 0.01$; ***$p < 0.001$.

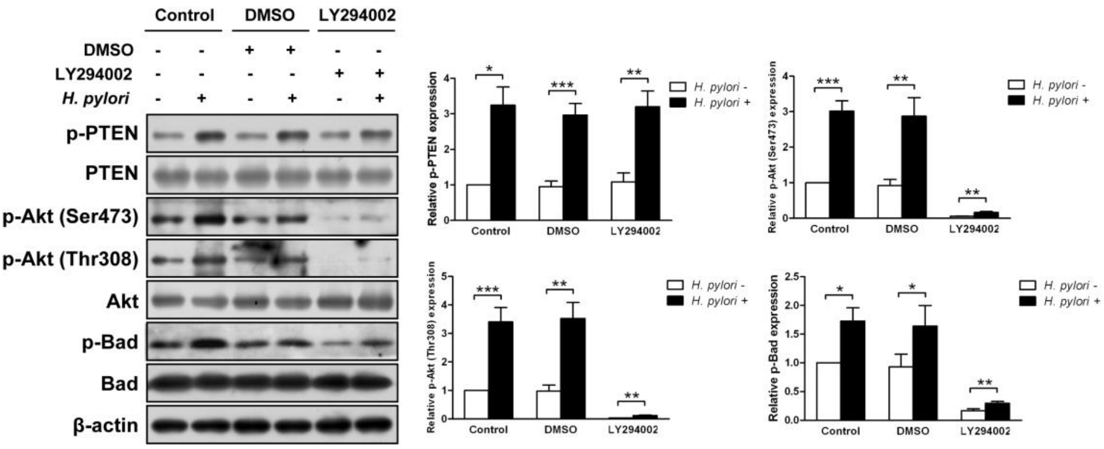

Figure S2. PI3K inhibition diminishes *H. pylori*-induced phosphorylation of the PI3K/Akt pathway. Immunoblots of phosphatase and tensin homolog/PI3K/Akt pathway-related proteins were used to quantify the relative protein expression levels (expressed as fold of control) in gastric epithelial cells infected with *H. pylori* for 1 h. Pharmacological inhibition of PI3K was achieved with LY294002 (40 μM pretreatment before incubation with *H. pylori*). The data are representative of three independent experiments. The samples derive from the same experiment and that blots were processed in parallel. *$p < 0.05$; **$p < 0.01$; ***$p < 0.001$.

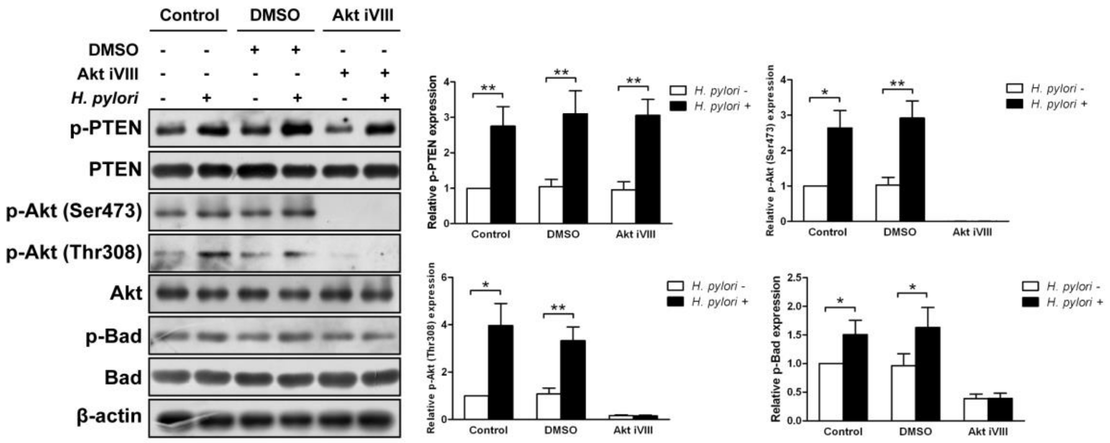

Figure S3. Akt inhibition blocks *H. pylori*-induced phosphorylation of the PI3K/Akt pathway. Immunoblots of phosphatase and tensin homolog/PI3K/Akt pathway-related proteins were used to quantify the relative protein expression levels (expressed as fold of control) in gastric epithelial cells infected with *H. pylori* for 1 h and treated with Akt iVIII (10 μM pretreatment before incubation with *H. pylori*). The data are representative of three independent experiments. The samples derive from the same experiment and that blots were processed in parallel. *$p < 0.05$; **$p < 0.01$.

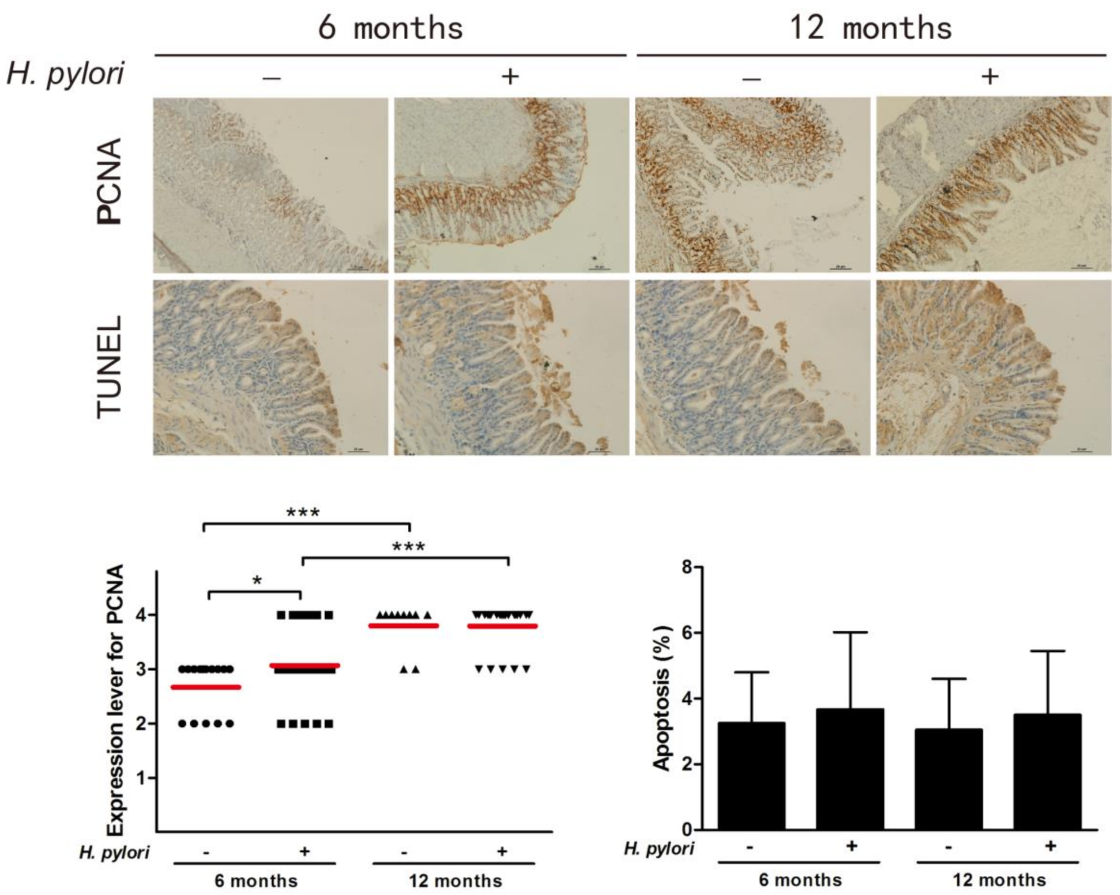

Figure S4. Proliferation and apoptosis in gastric tissue of Mongolian gerbils with *H. pylori* infection. Cells immunoreactive for proliferation cell nuclear antigen (PCNA) (ab29; 1:1800; Abcam, Cambridge, UK) in gastric tissue sections were semi-quantitatively assessed, and the protein expression levels are expressed as grade 1–4; mean grades (▬) for protein expression are shown. Apoptosis in tissue sections was determined by using a TUNEL assay, and the apoptosis rate was calculated as the ratio of TUNEL positive cells to the total cell number. Scale bar = 20 μm. *$p < 0.05$; ***$p < 0.001$.

Table S1 Association between phosphatase and tensin homolog (PTEN) and phosphorylated PTEN expression and clinicopathological data from gastric cancer patients

Characteristics	n	Overall grade of the protein expression									
		PTEN					p-PTEN				
		1	2	3	4	*P*	1	2	3	4	*P*
Gender											
Male	11	7	4	0	0	0.490	3	5	3	0	0.816
Female	14	8	3	2	1		5	5	4	0	
Age (years)											
≧55	10	7	2	0	1	0.485	2	6	2	0	0.813
<55	15	8	5	2	0		6	4	5	0	
Location											
Antrum	15	9	5	0	1	0.849	5	6	4	0	0.836
Body and cardia	10	6	2	2	0		3	4	3	0	
Gross type (Borrmann)											
Ⅰ+Ⅱ	2	1	1	0	0	0.909	1	1	0	0	0.394
Ⅲ +Ⅳ	23	14	6	2	1		7	9	7	0	
Differentiation											
Well and Moderately	12	9	3	0	0	0.093	2	7	3	0	0.434
Poorly and undifferentiatedly	13	6	4	2	1		6	3	4	0	
Invasive depth											
Above submucosa [1]	2	2	0	0	0	1 vs. 2: 1.000	0	1	1	0	1 vs. 2: 0.221
Muscularis propria [2]	2	2	0	0	0	1 vs. 3: 0.220	1	1	0	0	1 vs. 3: 0.354
Below subserosa [3]	21	11	7	2	1	2 vs. 3: 0.220	7	8	6	0	2 vs. 3: 0.450
TNM											
I + II	5	5	0	0	0	0.052	1	2	2	0	0.448
III + IV	20	10	7	2	1		7	8	5	0	
Lymph node metastasis											
+	19	11	6	2	0	0.884	4	9	6	0	0.097
-	6	4	1	0	1		4	1	1	0	

PTEN, phosphatase and tensin homolog; p-PTEN, phosphorylated PTEN

Table S2 Clinical characteristics of patients with different gastric lesions

Group	*n*	Sex		Age (mean ± SD)
		Male	Female	
Chronic non-atrophic gastritis	40	21	19	51.20 ± 14.07
Intestinal metaplasia	40	21	19	54.32 ± 12.18
Dysplasia	40	21	19	56.78 ± 11.81
Gastric carcinoma	40	21	19	53.82 ± 14.31
Overall	160	84	76	54.13 ± 13.05
